# Correction: The impact of maternal versus paternal imprisonment on their children’s health: A scoping review

**DOI:** 10.1371/journal.pone.0350225

**Published:** 2026-05-26

**Authors:** Naomi Gadian, Abigail Dunn, Donna Arrondelle, Sara Morgan, Paula Harriott, Lucy Wainwright, Emma Plugge

The third author’s name is spelled incorrectly. The correct name is: Donna Arrondelle. The correct citation is: Gadian N, Dunn A, Arrondelle D, Morgan S, Harriott P, Wainwright L, et al. (2025) The impact of maternal versus paternal imprisonment on their children’s health: A scoping review. PLoS One 20(7): e0329131. https://doi.org/10.1371/journal.pone.0329131.
