# Supplementary material for: The impact of maternal versus paternal imprisonment on their children’s health: A scoping review
Source: PLoS One. 2025 Jul 29;20(7):e0329131. doi: 10.1371/journal.pone.0329131 (PMC12306776; doi:10.1371/journal.pone.0329131)
Supplement: S1 File — (DOCX) - Appendix A -Search terminology and papers identified Appendix B - Table of Papers included and Table of Papers not included due to being reviews, however, their references were checked to ensure all relevant papers were included Appendix C - Explanation about the study populations for National Longitudinal Study of Adolescence Health (Add Health) and Fragile Families and Child Wellbeing Survey which were the two main studies Appendix D - Quality Assessment of the studies included, using NIH Quality Assessment Tool Appendix E - Funding assoicate with scoping review [file pone.0329131.s001.docx]

***Appendices***

**Appendix A**

Search terminology and papers identified

*Search terms*

Key search terms, including all languages with no timeframe limits, used were

- imprison* or incarcerat* or jail* or prison* or gaol*
- Child* or "young person" or adolescen* or teen* or youth*
- Maternal* or mother* or mum* or mom* or mam*
- paternal* or father* or dad* or papa*

(imprison* or incarcerat* or jail* or prison* or gaol*).mp. [mp=title, book title, abstract, original title, name of substance word, subject heading word, floating sub-heading word, keyword heading word, organism supplementary concept word, protocol supplementary concept word, rare disease supplementary concept word, unique identifier, synonyms]
(Child* or "young person" or adolescen* or teen* or youth*).mp. [mp=title, book title, abstract, original title, name of substance word, subject heading word, floating sub-heading word, keyword heading word, organism supplementary concept word, protocol supplementary concept word, rare disease supplementary concept word, unique identifier, synonyms]
(Maternal* or mother* or mum* or mom* or mam*).mp. [mp=title, book title, abstract, original title, name of substance word, subject heading word, floating sub-heading word, keyword heading word, organism supplementary concept word, protocol supplementary concept word, rare disease supplementary concept word, unique identifier, synonyms]
(paternal* or father* or dad* or papa*).mp. [mp=title, book title, abstract, original title, name of substance word, subject heading word, floating sub-heading word, keyword heading word, organism supplementary concept word, protocol supplementary concept word, rare disease supplementary concept word, unique identifier, synonyms]

*Papers identified*

Medline – 862->32 articles met the criteria (32 included)

CINHAL – 693 ->19 articles met the criteria (11 repeats so 8 included)

Psych Info – 1,650->24 articles met the criteria (10 repeats so 14 included)

EMBASE 2,806->25 articles met the criteria (20 repeats so 5 included)

Delphis 29-> 0 articles met the criteria

IBBS – search terms used anywhere except full text and after first 2,400 (no new ones between 1,000 and 2,400) – 40,260 -> 11 articles met the criteria (6 repeats so 5 included)

Web of Science - search terms above generated 427,478 after first 1,250 (no new ones between 1,000 and 1,250) 439,430 -> 29 (22 repeat so 7 included)

This totals 140 however, once duplicates were removed there were **71 papers**.

Of these 71 papers, a sample were jointly reviewed independently and agreement on their inclusion was reached through discussion if there were any uncertainties.

Ultimately **19 papers** have been included.

Following looking at the reference lists of the review papers, 1 additional paper was included.

This resulted in **20 papers being included.**

**Appendix B**

| Papers included | | | |
| --- | --- | --- | --- |
|  | Title | Author(s) | Year of publication |
| 1 | An Assessment of the Role of Parental Incarceration and Substance Misuse in Suicidal Planning of African American Youth and Young Adults. | Quinn CR, Beer OWJ, Boyd DT, Tirmazi T, Nebbitt V, Joe S | Journal of Racial & Ethnic Health Disparities. 9(3):1062-1074, 2022 Jun. |
| 2 | Assessing the relationship between parental imprisonment in childhood and risk of sexually transmitted infections: a cohort study of US adults in early adulthood. | Roettger M Houle B | BMJ Open. 11(4):e038445, 2021 04 01. |
| 3 | Bedtime Schedules and Sleep Regulation among Children of Incarcerated Parents. | Branigan AR Meyer JM | Journal of Pediatrics. 236:253-259, 2021 Sep. |
| 4 | Behavioral Reactions of Children to Parental Absence Due to Imprisonment | Fritsch T, Birkhead J. | Family Relations vol30 No1 pp83-88, 1981 |
|  | Child (un)awareness of parental incarceration as a risk factor: Evidence from South Korea | Woo, Y. and M. A. Kowalski | Journal of Child and Family Studies 2020 |
| 5 | C-Reactive Protein Levels Among U.S. Adults Exposed to Parental incarceration. | Boch SJ,  Ford JL | Biological Research for Nursing. 17(5):574-84, 2015 Oct. |
| 6 | Criminal Justice Involvement, Drug Use, and Depression Among African American Children of Incarcerated parents | Kopak, A. M. and D. Smith-Ruiz Race | and Justice 6(2): 89-116. |
| 7 | Early Onset of Sexual Intercourse and Parental Incarceration among African American Youth Living in Urban Public Housing. | Nebbitt V.E, Dexter R.V & Tirmaz M.T. | Journal of Urban Health 94(1): 125-135. 2017 |
| 8 | Health Care Use and Health Behaviours Among Young Adults With History of Parental Incarceration. | Heard-Garris N, Winkelman TNA, Choi H, Miller AK, Kan K Shlafer R, Davis MM | Pediatrics. 142(3), 2018 09. |
| 9 | Intergenerational Associations Between Parental Incarceration and Children's Sexual Risk Taking in Young Adulthood. | Le GT, Deardorff J, Lahiff M,  Harley KG | Journal of Adolescent Health. 64(3):398-404, 2019 03. |
| 10 | Maternal and paternal imprisonment in the stress process | Foster, H. and J. Hagan | Social Science Research 42(3): 650-669. |
| 11 | Parental incarceration and child mortality in Denmark. | Wildeman C, Andersen SH, Lee H, Karlson KB | American Journal of Public Health. 104(3):428-33, 2014 Mar. |
| 12 | Parental Incarceration and Child Overweight: Results From a Sample of Disadvantaged Children in the United States. | Branigan A.R., Wildeman C. | Public Health Reports. 134(4) (pp 363-370), 2019. Date of Publication: 01 Jul 2019. |
| 13 | Parental Incarceration and Child Sleep and Eating Behaviours. | Jackson DB, Vaughn MG | Journal of Pediatrics. 185:211-217, 2017 06. |
| 14 | Parental Incarceration and Child Well-being: Conceptual and Practical Concerns Regarding the Use of Propensity Scores. | Copp JE, Giordano PC, Manning WD, Longmore MA | Socius. 4, 2018 Jan-Feb. |
| 15 | Parental Incarceration and Child Wellbeing: Implications for Urban Families. | Geller A, Garfinkel I, Cooper CE, Mincy RB | Social Science Quarterly. 90(5):1186-1202, 2009 Dec 01. |
| 17 | Parental incarceration, depression and crime: An examination of incarceration timing, parental closeness and parent-child gender. | McDaniel, Chris Patrick | Dissertation Abstracts International Section A: Humanities and Social Sciences |
| 18 | Prisoners' assessments of mental health problems among their children. | Tasca M, Turanovic JJ, White C, Rodriguez N | International Journal of Offender Therapy & Comparative Criminology. 58(2):154-73, 2014 Feb. |
| 19 | The Impact of Parental Incarceration on Psychopathy, Crime, and Prison Violence in Women. | Thomson ND, Moeller FG Amstadter AB, Svikis D, Perera RA, Bjork JM | International Journal of Offender Therapy & Comparative Criminology. 64(10-11):1178-1194, 2020 08. |
| 20 | The Impact of Parental Incarceration on the Physical and Mental Health of Young Adults | Lee RD, Fang X, Luo F | Pediatrics. 131(4):e1188-95, 2013 Apr. |

| Papers not included as they were reviews, their references were checked | | | |
| --- | --- | --- | --- |
|  | Title | Author(s) | Year of publication |
| 1 | Punishment regimes and the multilevel effects of parental incarceration:  Intergenerational, intersectional, and interinstitutional models of social inequality and systemic exclusion | Foster, H. and J. Hagan | Annual  Review of Sociology 41: 135-158. 2015 |
| 2 | Post-traumatic stress disorder in prisoners' offspring: A systematic review and  meta-analysis | Gualtieri, G., et al. | Clinical Practice and Epidemiology in Mental Health 16: 36-45. 2020 |
| 3 | Children's antisocial behavior, mental health, drug use, and educational performance  after parental incarceration: a systematic review and meta-analysis | Murray, J., et al. | Psychological Bulletin 138(2): 175-210. 2012 |
| 4 | The effects of parental imprisonment on children. | Murray, J., et al. | 2008 |
| 5 | Parental incarceration, attachment and child psychopathology. | Murray J  Murray L | Attachment & Human Development. 12(4):289-309, 2010 Jul. |
| 6 | A Developmental Perspective on Children With Incarcerated Parents | Poehlmann-Tynan, J. and K. Turney | Child  Development Perspectives 15(1): 3-11. |
| 7 | Parental Incarceration and Children's Wellbeing | Turney, K. and R. Goodsell | Future of Children 28(1): 147-164. |
| 8 | Parental Incarceration and Child Health in the United States | Wildeman, C, Goldman AW, Turney K | Epidemiologic Reviews  40(1): 146-156. 2018 |

**Appendix C**

Explanation about study populations for National Longitudinal Study of Adolescence Health (Add Health) and Fragile Families and Child Wellbeing Survey

| **Study** | **National Longitudinal Study of Adolescence Health (Add Health)** | **Fragile Families and Child Wellbeing Survey** |
| --- | --- | --- |
| **Population** | Students randomly selected from schools that were nationally representative in the United States of America.  Students were included if they were in Grade 7 to 12 between 1994/5 and 2007/08. (80 high schools and 52 feeder schools stratified by school type, size, region, urbanicity, ethnic composition).  One study only included data relating to those who identified as African American and had information about parental incarceration.  90,118 students were initially surveyed in school using computer-assisted personal interview, with 20,745 asked to complete in-home surveys and had their weight and height taken.  Wave 4 was undertaken in 2008-09 when in home surveys were undertaken (15,701), along with their weight, height, waist, blood spot and urine samples. | 4,898 children born between 1998 and 2000 in 20 US cities.  Cities were chosen using stratified random stratified samples if they had populations greater than 200,000, hospitals. A sample of 75 hospitals were then taken from 20 cities, followed by random sample of both married and unmarried couples who agreed to participate.  There was purposeful oversampling of low-income, “unwed couples” (75% unmarried at the time of first interview).  Substantial number of parents had some experience of incarceration (42% of surveyed fathers and 7% surveyed mothers) by their child’s third birthday |
| **Data collection** | Wave 1 – April – December 1995 (response rate 79% of eligible participants from Wave 1)  Wave 2 – April -August 1996 (response rate 88.6%)  Wave 3 – August – April 2002 (response rate 77.4%)  Wave 4 – 2007-2008 (response rate 80.3%) | Parents /primary care giver were interviewed separately   - Wave 1 - within 24 hours of their child’s birth - Wave 2 - when their child was aged 1 year of age - Wave 3 - when their child was aged 3 years of age - Wave 4 - when their child was aged 5 years of age - Wave 5 – when their child was aged 9 years of age |
| **Exposure** | Participants were asked if their parents or parent figures had “ever spent time in jail or prison” | Participants were asked if they or their partner had a history of having spent time in jail or prison on or before the fourth wave of data collection and could be classified as maternal history, paternal history, both or neither. |
| **Outcomes** | Data was collected using multiple means, including face to face interviews at school and home with young people and separately with their parents/parent figures, blood spot tests and urine samples. | Data was collected using interviews with parents. |

**Appendix D**

Quality assessment of the studies included, using NIH Quality Assessment Tool

| **Title and authors** |  |  |  |  |  |  |  |
| --- | --- | --- | --- | --- | --- | --- | --- |
|  | **An Assessment of the Role of Parental Incarceration and Substance Misuse in Suicidal Planning of African American Youth and Young Adults, Quinn C; Beer O; Body D; Tirmazi T; Nebbitt V; Joe S.** | **Assessing the relationship between parental imprisonment in childhood and risk of sexually transmitted infections: a cohort study of US adults in early adulthood. Roettger M; Houle B** | **Bedtime Schedules and Sleep Regulation among Children of Incarcerated Parents, Branigan A, Meyer J** | **Child (Un)Awareness of Parental Incarceration as a Risk Factor: Evidence from South Korea, Woo Y; Kowalski M** | **C-Reactive Protein Levels Among U.S. Adults Exposed to Parental Incarceration. Boch S; Ford J.** | **Criminal Justice Involvement, Drug Use, and Depression Among African American Children of Incarcerated Parents. Kopak A, Smith-Ruiz D** | **Early Onset of Sexual Intercourse and Parental Incarceration among African American Youth Living in Urban Public Housing. Nebbitt V.E, Voisin D, Tirmazi M** |
| **Was the research question or objective in this paper clearly stated** | Y - investigating relations between parental problems including incarceration and suicidal planning among African American youth and young adults living in public housing in a large mid-Atlantic city in  the USA | Y - assesses whether parental imprisonment is a risk factor  for STIs and potential mediating factors that may explain  this association | Y – to investigate variation by parental incarceration history in the bedtime schedules set for elementary age children | Y - examine differences in developmental outcomes between children  cognizant of their parent’s incarceration and those who are unaware of parental imprisonment | Y – Analyse the association between low-grade inflammation and prior  biological parental incarceration | Y – Study to examine criminal justice involvement, drug use, and depressive symptoms in a large sample of African American children | Y -  explore factors impacting food insecurity in African American adolescents living in a public housing located in West Baltimor |
| **Was the study population clearly specified and defined** | Y | Y | Y - Fragile Families and Child Wellbeing Study  Population-based longitudinal family study using data from 5,000 children born in 20 US cities between 1998 and 2000. Parents were interviewed at birth, 1,3,5,9 years of age | Y | Y - National Longitudinal Study of Adolescent Health (Add Health) – United States | Y - National Longitudinal Study of Adolescent Health (Add Health) – United States | Y - |
| **Was the participation rate of eligible persons at least 50%** | CD – unclear how many were asked to contribute | N | CD - unclear how many were asked to contribute | Y | CD | CD | Y |
| **Were all the subjects selected or recruited from the same or similar populations (including the same time period), were inclusions and exclusion criteria for being in the study prespecified and applied uniformly to all participants?** | Y | Y | Y | Y | Y | Y | Y |
| **Was a sample size justification, power description, or variance and effect estimates provided** | Y | Y | Y | Y | Y | N | Y |
| **For the analyses in this paper, were the exposure(s) of interest measured prior to the outcomes(s) being measured** | CD – unclear order of questioning | CD – unclear order of questioning | CD - unclear order of questioning | CD | Y | CD | Y |
| **Was the timeframe sufficient so that one could reasonably expect to see an association between exposure and outcome if it existed** | CD – unclear if the timing of parental imprisonment was factored in analysis | Y | Y | Y | Y | Y | Y |
| **For exposred that can vary in amount or level, did the story examine different levels of the exposure as related to the outcome (e.g. categories of exposure, or exposure measured as continuous variable)?** | N | N | Y | NA | Y | Y | NA |
| **Were the exposure measures (independent variables) clearly defined, valid, reliable and implemented consistently across all study participants?** | Y | Y | Part - clear defined but self reported | partly - self reported | partly - self reported | partial - self reported | Partial - self reported |
| **Was the exposure (s) assessed more than once over time?** | N | N | Y | CD | N | Y | N |
| **Were the outcome measures (dependent variables) clearly defined, valid, reliable, and implemented consistently across all study participants?** | Y | Y | Part - clearly defined but self reported | partly - self reported | Y | Y | Partial - self reported |
| **Were the outcome assessors blinded to the exposure status of participants?** | N | CD | CD | CD | CD | CD | CD |
| **Was loss to follow-up after baseline 20% or less?** | CD – unclear original number asked | CD | CD | Y | CD | CD | Y |
| **Were key potential confounding variables measured and adjusted statistically for their impact on the relationship between exposure(s) and outcome(s)?** | N – alcohol, drug, drug | Y – respondent age and parent age incarceration, ethnicity, sex, physical abuse, parent education, family poverty, BMI, school attachment, drugs and alcohol, STI | Y - 1) individual factors,  (2)  family characteristics,  (3)  geography, | Y – sociodemographics, family relations and living arrangements. | Y – socioeconomic demographics, education, health and wellbeing, | Y – age, education, employment, | N |
|  | Good/Fair | Good | Good – including explanation of limitations | Good | Good/Fair clear explanation and analysis but practical implications unclear | Fair/Good | Good/Fair – Did discuss potential bias and limitations but limited confounders |

| **Title and authors** |  |  |  |  |  |  |  |
| --- | --- | --- | --- | --- | --- | --- | --- |
|  | **Health Care Use and Health Behaviours Among Young Adults with History of Parental Incarceration. Heard-Garris N, Winkelamn T, Choi H, Mikller A, Kan K, Shlafer R, Davis M.** | **Intergenerational Associations Between Parental Incarceration and Children’s Sexual Risk Taking in Young Adulthood. Le G; Deardorff J; Lahiff M; Harley K.** | **Maternal and paternal imprisonment in the stress process . Foster H, Hagan J** | **Parental Incarceration and Child Mortality in Denmark. Wildeman C, Anderson S, Lee Hedwig, Karison K** | **Parental Incarceration and Child Sleep and Eating Behaviors. Jackson D, Vaughn M** | **Parental Incarceration and Child Wellbeing: Implications for Urban Families. Geller A, Garfinkel I, Cooper C, Mincy R.** | **Behavioral Reactions of Children to Parental Absence due to Imprisonment. Fritsch T, Burkhead J.** |
| **Was the research question or objective in this paper clearly stated** | Y - examine associations between history of mother incarceration (MI) and father incarceration (FI) | Y - Estimate the associations between parental incarceration and sexual risk outcomes (early sexual onset, inconsistent condom use, and sexually transmitted infections (STIs) in young adulthood | Y - Iinvestigate the influences of maternal and paternal imprisonment on changes in young  adult mental health using a nationally representative sample. | Y - Use Danish registry data to examine the association between  parental incarceration and child mortality risk | Y - examine whether parental incarceration is significantly associated with a number of sleep and eating  behaviors among offspring during early childhood | Y - examining the economic, residential, and developmental risks posed by parental incarceration | Y - ? Do children  exhibit different reactions depending on which parent is committed to prison? |
| **Was the study population clearly specified and defined** | Y - National Longitudinal Study of Adolescent Health (Add Health) – United States | Y - National Longitudinal Study of Adolescent Health (Add Health) – United States | Y - National Longitudinal Study of Adolescent Health (Add Health) – United States | Y | Y - Fragile Families and Child Wellbeing Study  Population-based longitudinal family study using data from 5,000 children born in 20 US cities between 1998 and 2000. Parents were interviewed at birth, 1,3,5,9 years of age | Y - Fragile Families and Child Wellbeing Study  Population-based longitudinal family study using data from 5,000 children born in 20 US cities between 1998 and 2000. Parents were interviewed at birth, 1,3,5,9 years of age | Y |
| **Was the participation rate of eligible persons at least 50%** | CD | CD | CD | Y | CD | CD | Y |
| **Were all the subjects selected or recruited from the same or similar populations (including the same time period), were inclusions and exclusion criteria for being in the study prespecified and applied uniformly to all participants?** | Y | Y | Y | Y | Y | Y | Y |
| **Was a sample size justification, power description, or variance and effect estimates provided** | Y | Y | Y | Y | Y | y | N |
| **For the analyses in this paper, were the exposure(s) of interest measured prior to the outcomes(s) being measured** | CD | CD | CD | Y | CD | y | N |
| **Was the timeframe sufficient so that one could reasonably expect to see an association between exposure and outcome if it existed** | Y | Y | Y | Y | Y | y | Y |
| **For exposures that can vary in amount or level, did the story examine different levels of the exposure as related to the outcome (e.g. categories of exposure, or exposure measured as continuous variable)?** | N | N | N | Y | Y | y | N |
| **Were the exposure measures (independent variables) clearly defined, valid, reliable and implemented consistently across all study participants?** | Y | Y | Y | Y | Y | y | Y |
| **Was the exposure (s) assessed more than once over time?** | N | N | Y | Y | Y | Y | N |
| **Were the outcome measures (dependent variables) clearly defined, valid, reliable, and implemented consistently across all study participants?** | Y Self-reported | Y partial- self reported except for STI which was diagnosed by urine sample | partial - self reported | Y | Y partial - parent reported | Y | Partial |
| **Were the outcome assessors blinded to the exposure status of participants?** | CD | CD | CD | Y | N | CD | CD |
| **Was loss to follow-up after baseline 20% or less?** | CD | CD | N | Y | CD | Y | N |
| **Were key potential confounding variables measured and adjusted statistically for their impact on the relationship between exposure(s) and outcome(s)?** | Y - demographics, education, geographical area | Y | Y | Y – socio-demographics, age, | Y - age, race, sex, maternal education, household income, maternal depression, | Y – race, ethnicity, age, education | N |
|  | Good – limitations clearly discussed | Good – limitations clearly discussed | Fair | Good | Good – with some limitations | Good | Fair – limited study |

| **Title and authors** |  |  |  |  |  |  |
| --- | --- | --- | --- | --- | --- | --- |
|  | **Parental Incarceration, Depression and Crime: An Examination of Incarceration Timing, Parental Closeness and Parent-Child Gender. McDaniel** | **Prisoners’ Assessments of Mental Health Problems Among Their Children. Tasca M, Turanovic J., White C. Rodriguez N.** | **The Impact of Parental Incarceration on Psychopathy, Crime, and Prison Violence in Women. Thomson N, Moeller F, Amstadter A, Svikis D, Perera R, Bjork J.** | **The Impact of Parental Incarceration on the Physical and Mental Health of Young Adults. Lee R, Fang X, Luo F.** | **Parental Incarceration and Child Overweight: Results From a Sample of Disadvantaged Children in the United States. Branigan A, Wildeman C.** | **Parental Incarceration and Child Wellbeing: Conceptual and Practical Concerns Regarding the use of propensity scores by Copp, Giordano, Manning, Longmore** |
| **Was the research question or objective in this paper clearly stated** | N – part of a wider dissertation | Y - Assess whether incarcerated mothers are more likely than incarcerated fathers to report t hat their children are experiencing mental health problems | Y – Association between childhood exposure to paternal and/or maternal incarceration on adulthood psychopathic traits, criminal offending, and prospective prison violence over 12 months | Y – Investigate the relationship between parental incarceration history and young adult physical and mental health outcomes | Y - Examine whether parental incarceration was associated with child overweight at age 9 and whether that association differed by which parent was incarcerated. | Y - examine the appropriateness of propensity score methods for the study of incarceration effects on children by directing attention to a range of conceptual and practical concerns, including the exclusion of theoretically meaningful covariates, the comparability of treatment and control groups, and potential ambiguities resulting from researcher-driven analytic decisions |
| **Was the study population clearly specified and defined** | Y  National Longitudinal Study of Adolescent Health (Add Health) – United States | y | y | Y | Y - Fragile Families and Child Wellbeing Study  Population-based longitudinal family study using data from 5,000 children born in 20 US cities between 1998 and 2000. Parents were interviewed at birth, 1,3,5,9 years of age | Y - Fragile Families and Child Wellbeing Study  Population-based longitudinal family study using data from 5,000 children born in 20 US cities between 1998 and 2000. Parents were interviewed at birth, 1,3,5,9 years of age |
| **Was the participation rate of eligible persons at least 50%** | CD | y | CD | Y | CD | CD |
| **Were all the subjects selected or recruited from the same or similar populations (including the same time period), were inclusions and exclusion criteria for being in the study prespecified and applied uniformly to all participants?** | Y | y | Y | Y | Y | CD |
| **Was a sample size justification, power description, or variance and effect estimates provided** | N | n | N | Y | Y | N |
| **For the analyses in this paper, were the exposure(s) of interest measured prior to the outcomes(s) being measured** | CD | cd | CD | CD | CD | CD |
| **Was the timeframe sufficient so that one could reasonably expect to see an association between exposure and outcome if it existed** | Y | N | Y | Y | Y | CD |
| **For exposure that can vary in amount or level, did the story examine different levels of the exposure as related to the outcome (e.g. categories of exposure, or exposure measured as continuous variable)?** | Y | na | N | Y | Y | CD |
| **Were the exposure measures (independent variables) clearly defined, valid, reliable and implemented consistently across all study participants?** | Y | y | Y | Y | partial - self reported but corroborated where possible with other parent | CD |
| **Was the exposure (s) assessed more than once over time?** | Y | n | N | N | Y | CD |
| **Were the outcome measures (dependent variables) clearly defined, valid, reliable, and implemented consistently across all study participants?** | Y | N/partial - parent reported | Y | Yes -they need to be told by health care provided/doctor however, still self reported | Y | Y |
| **Were the outcome assessors blinded to the exposure status of participants?** | CD | N | N | N | CD | CD |
| **Was loss to follow-up after baseline 20% or less?** | CD | Y | NA | N | CD | CD |
| **Were key potential confounding variables measured and adjusted statistically for their impact on the relationship between exposure(s) and outcome(s)?** | Y | N | N | Y – sociodemographic, family structure, education, alcohol, maltreatment | Y – sociodemographic, maternal obesity, impulsivity, child temperament | Y |
|  | Fair with limitations | Fair /poor | Fair | Good with limitations | Good- with some limitations | Fair |

**Appendix E**

Funding: There was no specific funding associated with this scoping review however, the lead author was funded as part of the NHS England training programme

Data Avaliability: All data avaliable through published journals
