# Supplementary material for: The impact of maternal versus paternal imprisonment on their children’s health: A scoping review
Source: PLoS One. 2025 Jul 29;20(7):e0329131. doi: 10.1371/journal.pone.0329131 (PMC12306776; doi:10.1371/journal.pone.0329131)
Supplement: S2 File — (DOCX) All data was avaliable through published journals [file pone.0329131.s002.docx]

Dear Editorial Team

I have received a request for further detail about my manuscript's Data Availability statement and minimal data set. You kindly supported me with what was required including In the context of a scoping review, a require the minimal dataset, including the same information generally included in meta-analyses and systematic reviews. Specifically, this should include the search strategy you employed and any eligibility/exclusion criteria.

The search strategy, exclusion and inclusion criteria are stated in the report (Table 1 in the report) and include below.

Thank you for your help with this

Best wishes

Naomi Gadian

Search terminology and papers identified

*Search terms*

Key search terms, including all languages with no timeframe limits, used were

- imprison* or incarcerat* or jail* or prison* or gaol*
- Child* or "young person" or adolescen* or teen* or youth*
- Maternal* or mother* or mum* or mom* or mam*
- paternal* or father* or dad* or papa*

(imprison* or incarcerat* or jail* or prison* or gaol*).mp. [mp=title, book title, abstract, original title, name of substance word, subject heading word, floating sub-heading word, keyword heading word, organism supplementary concept word, protocol supplementary concept word, rare disease supplementary concept word, unique identifier, synonyms]
(Child* or "young person" or adolescen* or teen* or youth*).mp. [mp=title, book title, abstract, original title, name of substance word, subject heading word, floating sub-heading word, keyword heading word, organism supplementary concept word, protocol supplementary concept word, rare disease supplementary concept word, unique identifier, synonyms]
(Maternal* or mother* or mum* or mom* or mam*).mp. [mp=title, book title, abstract, original title, name of substance word, subject heading word, floating sub-heading word, keyword heading word, organism supplementary concept word, protocol supplementary concept word, rare disease supplementary concept word, unique identifier, synonyms]
(paternal* or father* or dad* or papa*).mp. [mp=title, book title, abstract, original title, name of substance word, subject heading word, floating sub-heading word, keyword heading word, organism supplementary concept word, protocol supplementary concept word, rare disease supplementary concept word, unique identifier, synonyms]

Table 1. Study Inclusion and exclusion criteria

| **Inclusion** | | **Exclusion** |
| --- | --- | --- |
| **Participants** | Any offspring of imprisoned mothers and fathers | If parent was detained in immigration removal centres or secure psychiatric units or was a prisoner of war |
|  | Male or Female |  |
| **Intervention** | Experience of maternal imprisonment or father imprisonment, or both, within the same study population.  The comparison group may be experiencing no parental imprisonment. | Only focused on maternal or only paternal imprisonment |
| **Outcome** | Any health condition including:   - Physical health - Mental health - Behavioural health, for example behavioural challenges and sleeping.   The paper must report health outcomes for children who have experienced maternal imprisonment and children who have experienced paternal imprisonment. | Not a health condition, such as   - income, - homeless, - school achievement - educational level - sporting achievement - involvement in the criminal justice system - family relations |
| **Study** | Prevalence studies, cross-sectional studies, cohort studies, case control studies, surveys, | Policy, opinion, review articles.  (Whilst review articles were excluded, the reference lists were reviewed to check for any missed papers.) |
|  | Any country, any date of publication, any language |  |
|  | Published and un-published data |  |
