# Supplementary material for: The impact of maternal versus paternal imprisonment on their children’s health: A scoping review
Source: PLoS One. 2025 Jul 29;20(7):e0329131. doi: 10.1371/journal.pone.0329131 (PMC12306776; doi:10.1371/journal.pone.0329131)
Supplement: Attachment — Submitted filename: impact maternal v paternal imprisonment_SM.docx [file pone.0329131.s003.docx]

The impact of maternal versus paternal imprisonment on their children’s health: A scoping review

**General Comments:**

Thank you for the opportunity to review this manuscript. This topic is critical for the improvement and understanding of broader family and more specifically child mental health and general wellbeing. I am glad to see more research comparing the effects of maternal versus paternal factors on offspring general and mental health. I believe this paper has the potential to be a meta-analysis, although I understand and appreciate the level of additional work and time that may prohibit this endeavour. However, it may provide more depth to your findings. Overall, I think this manuscript has the potential to contribute greatly to the existing literature, although there are a number of points that need to be addressed:

It would be useful to know the age of the children you are referring to in the manuscript. Did you solely look at anyone who had a parent incarcerated, or children and adolescents (e.g. 0-18)? It may be helpful for the reader to see this represented in your tables to understand the context of health and wellbeing problems due to timing of exposure to this form of ACE. This is particularly relevant when comparing studies with the same health outcomes but different populations. Consider also adding the sex/gender of those children included in each study, to help readers understand whether there may be sex differences in these effects. It may also help determine generalisability of findings.

It would also be worth mentioning, if available, the average incarceration period. Is it possible that fathers are incarcerated for longer periods and the effects of paternal incarceration is therefore different. It is difficult to infer anything tangible when we may be comparing vastly different prison terms. Either consider adding to tables if available, or consider adding as a limitation or future direction for ongoing research.
(for context, a mother incarcerated for 4 years versus a father incarcerated for 6 months may have profoundly different effects on the child).

Consistency of language throughout - for example, wellbeing versus well-being are used.

There are inconsistencies with double spacing between words throughout the manuscript, please revise.

Make sure tense is consistent throughout the manuscript, i.e. examine versus examined; are based versus were based; analyse versus analysed.

The statistics represented in your tables are a bit confusing. Some refer to 1.6 times. Others represent what I presume are r scores. You have also used two different forms of parentheses (e.g. ( and [ ) in these tables, and I am unsure if that is due to differences in findings or a reporting error. You also report confidence intervals and I think standard errors, dependent on the study. Is there a reason for this? You have also omitted the interpretation of SE in your footnotes for these tables.

The tables are also not in APA format. There are a number of formatting, referencing, grammatical and punctuation errors throughout that require attention. I have done my best to make more specific comments below that may help in this process, although I have likely missed some and gently recommend your attention with a more fine grained approach. I understand this is sometimes challenging with multiple authors, so suggest the primary author to choose a tense, and referencing style, and go from there. Some examples are headings/bolding and italicising of text, table names and numbers.

I also wonder why you have listed the statistics in the paragraphs and reiterated them again in the tables. This method is viewed unfavourably by reviewers and readers as it increases burden. I would instead suggest referring to the findings broadly in text, and referring to the tables for statistics. E.g. There were inconsistent results relating to …. for children/adolescents/offspring experiencing MI versus FI.. where X studies identified increased ….., while X studies identified decreased…. and X studies identified no association between Y and Z (see Table X).
You also can probably omit the authors name in that section as you list them in the table, this will shorten the manuscript and it will sound much more concise (and help interpretability of findings).

**Specific Comments:**

Line 9: ‘...wellbeing outcomes who children who experience…’ I think you may have meant to say wellbeing outcomes *for* children who..

Line 27: ‘Having either parent in prion..’ Correct spelling error, prison.

Line 52: Consider caregiving responsibility rather than caring responsibility

Line 69: ‘...being unable to see or talk can be extensive…’ - revise sentence, important to be specific. E.g. ‘unable to see or talk’ in this context would refer to seeing or talking to their incarcerated parent, not unable to see or talk generally.

Line 75: unnecessary use of :

Line 79: Omit ‘statistically’

Line 80: Be specific, significantly associated with PTSD/mental health conditions for whom? The incarcerated parent or their offspring?

Line 94: revise this sentence. ‘..be taken into statutory care of grandparents’ is an incomplete sentence. E.g. ‘be taken into statutory or grandparental care’

Line 95-96: Revise sentence and use of ‘individual separate’

Line 112: ‘process: included’ should this be ‘process included:’ ?

Line 143: Table 1 - Use of various font sizes, inconsistent punctuation. Repeated mention of checking review articles’ reference list.

Line 158-159: Check APA guidelines for use of written versus numerics for numbers 10 and above. E.g. sixty-nine versus 71

Line 177: Counties or countries?
Line 177: mortality of children or the incarcerated parent?

Line 178: Parental reports of depression of their children or themselves?

Line 186: Revise this sentence. United States of American Adolescents.. ?

Line 190: Is the 15701 the sample at wave 4? If yes, indicate with *n=*

Line 191-192: You mention ‘in jail’ or ‘in prison’. If there is a difference in jail versus prison, you should elaborate in the introduction/method. If there is not a difference and you want to capture both terms, use incarcerated or imprisoned, just to not confuse the reader

Line 210-214: You have previously established MI versus FI, so use those terms consistently. You begin discussing no parental imprisonment earlier in the manuscript, so it would be better to define this term (NPI) earlier and use consistency throughout.

Line 218: Make sure statistics are represented in APA format (i.e. italicised where required)

Line 234: ‘AddHealth’ should there be a space?

Line 235: Revise sentence ‘as demonstrated by two separate studies showed that’

Line 238: ‘Geller et al’ - this is not APA and it’s one of the first times you have mentioned et al. with an intext reference. Please ensure you have this consistent throughout, usually in-text references also require publication year in parenthesis. You also inconsistency use a period after et al. throughout this and preceding and succeeding paragraphs.

Line 252: use of experienced and experience in the same sentence. Recommend going through the paper and correcting all to be in past tense.

Line 254: may be helpful to define ‘acting-in’ - I presume this opposes acting-out, but may be helpful to clarify

Table 3: Please check the statistics you are representing. Some of the CI’s have a zero before the period, some do not. Some of the papers have the statistic represented in parenthesis (e.g. CI or SE) while others do not, yet still have statistics reported. Please ensure you are determining the statistical test you are referencing. It is difficult to compare across studies with different statistical tests represented, so ensure you are being clear about what is reported and list all of the terms in the footnote (e.g. t-test, OR, SE, Beta)

It is also unclear whether you are referring to reports on offspring. For example, parent self-report suggests that the mother or father is reporting on their own mental health experience, whereas using parent-report would indicate their report on their child. Self-report suggests child reporting on their own mental health etc.

Line 280: There is a sentence that is in parenthesis but is separated from the previous sentence by a period.

Line 303-305: statistics need additional parentheses and parenthesis within a parenthesis requires use of square brackets: i.e. (aOR 1.7, 95% CI[1.1−2.8]). Statistics listed in line 304 are not in parenthesis while those in line 305 are. This occurs again on line 309.

Line 306: You did not define STI in the above line before using the abbreviation.

Table 4: Is there a difference between the reference to ‘use’ versus ‘abuse’. In this table you indicate cocaine, marijuana, or substance use, and then prescription drug abuse.

Also maybe move the Table 4 outcomes to under the substance and alcohol use section, and keep Table 5 under diet, etc.

Line 335: ‘Indeed…..same study population’. Revise this sentence.

Line 353: ‘experiencing parental imprisonment…impact their children’s health and wellbeing’. If you are referring to the *child* experiencing parental imprisonment it impacts *their* health and wellbeing, not their children’s.

Line 356-357: Reference?

Line 363: atomised? Maybe inconsistent or incongruent?

Line 364: reviewing the impact maternal and paternal imprisonment on children. *Has* on children? The impact *of* maternal and paternal… etc. Revise

Line 364-365: I think this is implied. You could instead say, ‘this is a first attempt to synthesise existing literature relating to maternal and paternal imprisonment to gain a holistic understanding of the parental-gender impacts of incarceration on their child, both physically and psychologically’. This wasn’t an attempt, you did a great job with the literature you had - ensure you emphasise that this highlights an existing deficit within the literature that has failed to examine the potential risk for child health outcomes dependent on whether they experience a mother or father caregivers incarceration, compared to non-imprisoned parents.

Line 366: Very casual language. Revise. Avoid saying ‘very variable’ or ‘from how’. Use terms like methodological limitations that precluded meaningful interpretation, or a lack of consistency in definitions and measurements throughout the literature pertaining to parental incarceration and child outcomes.

Line 363-381: This section is a bit repetitive. Start by outlining the issues broadly, then use a sentence for each concern to specify the issue and why it is an important limitation for your study. Don’t be afraid to break limitations down to themes, i.e. ‘Methodological limitations’, ‘Measurement outcome limitations’, etc.

Line 382-388: Parent-reports or child mental health are also extremely incongruent to child self-reported mental health. There is a lot of subjectivity to consider, if a parent experiences their own mental health issue/substance or alcohol abuse issue, they are less likely to be acutely aware of physical or mental health issues in their child. Further, depression, anxiety, self harm and suicidality are extremely challenging for parents to be cognisant of, which adds another level of subjectivity and bias. There may also be a bit of social desirability bias, where parents where one or more caregivers have experienced imprisonment may want to avoid sharing the depth of detrimental impacts on their children, and report more favourable outcomes. These are just some considerations.

Line 420: Consider another possibility that hypersexuality is rarely considered for youth as often there is a limited understanding of consent. It is possible that there is inconsistency in the age of investigation in early sexual behaviour due to the grey area between early sexuality and sexual abuse. Try your best when critiquing the inconsistency in the literature to propose other possibilities rather than methodological differences, which you have already touched on. Instead consider social and environmental impacts, as well as age of consent, lack of boundary setting from parents who have been incarcerated, poor parental monitoring, externalising behaviours, etc.

Line 451: You say you have limited understanding of the implications, but then say despite the substantial implications. Are you referring to the mechanism by which parental imprisonment is implicated in poorer mental health and physical wellbeing. If yes, say that explicitly.

Line 456: Reference?

Line 458-461: This is a bit of a scoping claim. Make sure you support it with references, or if it is your opinion, say ‘although this interpretation is speculative’ or something along those lines.

Line 464: A lot of what you state in the strengths and weaknesses have been mentioned earlier in the discussion. Make sure you synthesise the discussion to ensure it reads better and is less repetitive.

Line 511-518: I think your conclusion is lacking the overarching take away. While it is evident that parental incarceration may place children at risk of detrimental physical and psychological outcomes, there is still limited understanding of the differences that may occur when it is a mother versus a father that is imprisoned. Further research is necessary to understand the potential similarities and differences based on parental sex, and the influence this has on child functioning … etc
