# Supplementary material for: The impact of maternal versus paternal imprisonment on their children’s health: A scoping review
Source: PLoS One. 2025 Jul 29;20(7):e0329131. doi: 10.1371/journal.pone.0329131 (PMC12306776; doi:10.1371/journal.pone.0329131)
Supplement: Attachment — Submitted filename: Response to reviewers.docx [file pone.0329131.s005.docx]

Dear Reviewers,

We are grateful to the reviewers for their thoughtful comments. We included the comments into one word document below. We have used this document to methodically describe how we have addressed each of the issues they raise. Our responses are written in red.

We have ensured that grammatical, punctuation, referencing, sentence structure and content has been revised as addressed in the reviewers comments.

Thank you for your time reviewing this submission

Yours Sincerely

Reviewers' comments:

Reviewer's Responses to Questions

**Comments to the Author**

1. Is the manuscript technically sound, and do the data support the conclusions?

The manuscript must describe a technically sound piece of scientific research with data that supports the conclusions. Experiments must have been conducted rigorously, with appropriate controls, replication, and sample sizes. The conclusions must be drawn appropriately based on the data presented.

Reviewer #1: Partly

Reviewer #2: Yes

2. Has the statistical analysis been performed appropriately and rigorously?

Reviewer #1: Yes

Reviewer #2: N/A

3. Have the authors made all data underlying the findings in their manuscript fully available?

The [PLOS Data policy](https://gbr01.safelinks.protection.outlook.com/?url=http%3A%2F%2Ftrack.editorialmanager.com%2FCL0%2Fhttp%3A%252F%252Fwww.plosone.org%252Fstatic%252Fpolicies.action%2523sharing%2F1%2F010f0193d923f10e-13e96bce-e92f-410b-b4b9-2b5ea34a3e5a-000000%2FmX_s-K4hoZugh4PRxZbWJJLC1yPmtuoae06Jr-FeaZ0%3D189&data=05%7C02%7Cnaomi.gadian%40nhs.net%7C5e94276ab36d4965c8de08dd1f48048c%7C37c354b285b047f5b22207b48d774ee3%7C0%7C1%7C638701116624545620%7CUnknown%7CTWFpbGZsb3d8eyJFbXB0eU1hcGkiOnRydWUsIlYiOiIwLjAuMDAwMCIsIlAiOiJXaW4zMiIsIkFOIjoiTWFpbCIsIldUIjoyfQ%3D%3D%7C0%7C%7C%7C&sdata=CelYA%2Fj7TyFWjcybJYk1W4iXZfaenvxCaMrnagyJ5V0%3D&reserved=0) requires authors to make all data underlying the findings described in their manuscript fully available without restriction, with rare exception (please refer to the Data Availability Statement in the manuscript PDF file). The data should be provided as part of the manuscript or its supporting information, or deposited to a public repository. For example, in addition to summary statistics, the data points behind means, medians and variance measures should be available. If there are restrictions on publicly sharing data—e.g. participant privacy or use of data from a third party—those must be specified.

Reviewer #1: Yes

Reviewer #2: Yes

4. Is the manuscript presented in an intelligible fashion and written in standard English?

PLOS ONE does not copyedit accepted manuscripts, so the language in submitted articles must be clear, correct, and unambiguous. Any typographical or grammatical errors should be corrected at revision, so please note any specific errors here.

Reviewer #1: Yes

Reviewer #2: Yes

5. Review Comments to the Author

Please use the space provided to explain your answers to the questions above. You may also include additional comments for the author, including concerns about dual publication, research ethics, or publication ethics. (Please upload your review as an attachment if it exceeds 20,000 characters)

Reviewer #1: This is an important topic that is worthy of investigation. While the author has done well to synthesise a confusing and methodologically inconsistent body of literature, there are a number of important grammatical, punctuation, referencing, sentence structure and general content issues that require attention. The content however, is sound. I have attached a number of comments which I hope will help in the resubmission of this article. Well done authors.

Thank you for your comments, we have addressed your recommendations.

Reviewer #2: Thank you for the opportunity to review this timely, useful, and well-written review. I have a few tiny comments.

Thank you for your comments, we have addressed your recommendations.

Abstract is clear and succinct.
Typo in abstract “prion” This has been changed to prison
Intro
Page 2- “ACEs” not “ACE’s” This has been changed to ACEs
“offspring” is an odd choice of words. Can you use “children”? Thank you, this has been changed in the report to use children.

Can you comment on whether it matters that children are biological or not? Studies did not review this and studies asked if parents were imprisoned but not if they were biological or not. We have therefore highlighted this in the discussion section (line 516).
Methods
Research question- I suggest removing “well being.” Later you clarify you only mean health conditions, not things like poverty, so it would be clearer to just say health. Wellbeing has been removed throughout the manuscript.
Identifying studies- please include the search terms.

The search terms are included in Appendix A and a reference to this has been added in the text
Study selection- You introduce quality assessment on page 4 but only explain it on page 6. I suggest removing the mention of quality assessment until that subsection on page 6. Quality assessment is not strictly necessary for scoping reviews.

Added to Page 6 as per comment.
Page 7- is “unwed” the term used in the survey itself? An anachronistic term.

The study used this terminology and has been put in “” to demonstrate this

Page 10- that that typo This has been changed.
Discussion
The discussion is meandering and includes some information better placed in the intro (e.g. challenges with visits). I recommend paring it down and focusing on health outcomes, as these are all your review included (e.g. is “confusion” or “resentment” a health outcome?).

We have restructured and reduced the word count of the discussion. It is now more concise and focused. Material has been moved to the introductory section as suggested above.

Page 20- Isn’t it Nebbit et al. who define early sex as under 13? –This has been changed to ensure that the correct studies are referenced.
Line 423- parents’ needs an apostrophe - This has been changed and an apostrophe added.
Line 429-431: remove, not part of your question - This has been changed.
Line 460 ‘gender of parental imprisonment”- do you mean gender of imprisoned parent? This has been changed to gender of imprisoned parent.
Line 488: “None of the studies commented on consideration for same sex parenting.”- thank you for acknowledging this, and you could add that the LGTBQ+ community is hyper-policed and hyper-incarcerated, and how this may therefore be particularly relevant for the children of incarcerated people.

The reviewer makes an important point and we have added to the sentence, ‘although LGBTQI+ individuals are over-represented in prison populations’.

Reviewer 1

The impact of maternal versus paternal imprisonment on their children’s health: A scoping review

General Comments:

Thank you for the opportunity to review this manuscript. This topic is critical for the improvement and understanding of broader family and more specifically child mental health and general wellbeing. I am glad to see more research comparing the effects of maternal versus paternal factors on offspring general and mental health. I believe this paper has the potential to be a meta-analysis, although I understand and appreciate the level of additional work and time that may prohibit this endeavour.

Unfortunately the studies measure different outcomes and even when the studies do measure the same thing they use a different tool therefore meta-analysis would not be appropriate.

However, it may provide more depth to your findings. Overall, I think this manuscript has the potential to contribute greatly to the existing literature, although there are a number of points that need to be addressed:

It would be useful to know the age of the children you are referring to in the manuscript. Did you solely look at anyone who had a parent incarcerated, or children and adolescents (e.g. 0-18)? It may be helpful for the reader to see this represented in your tables to understand the context of health and wellbeing problems due to timing of exposure to this form of ACE. This is particularly relevant when comparing studies with the same health outcomes but different populations.

We included studies that studied anyone who as a child/adolescence aged 0-18 years experienced their parent being incarcerated. The age of the experience/children included was not detailed in the included studies apart from being child/adolescence. We have made this clear in the text in line 136 and 173. This information is also captured in Table 1.

Consider also adding the sex/gender of those children included in each study, to help readers understand whether there may be sex differences in these effects. It may also help determine generalisability of findings.

Unfortunately, this was not often available – where it is available this has been included e.g. Table 2

It would also be worth mentioning, if available, the average incarceration period. Is it possible that fathers are incarcerated for longer periods and the effects of paternal incarceration is therefore different. It is difficult to infer anything tangible when we may be comparing vastly different prison terms. Either consider adding to tables if available, or consider adding as a limitation or future direction for ongoing research. (for context, a mother incarcerated for 4 years versus a father incarcerated for 6 months may have profoundly different effects on the child).

Unfortunately, the length of incarceration data was not available in the studies and so could not be included.

Consistency of language throughout - for example, wellbeing versus well-being are used.

There are inconsistencies with double spacing between words throughout the manuscript, please revise.

We have reviewed and addressed your recommendations.

Make sure tense is consistent throughout the manuscript, i.e. examine versus examined; are based versus were based; analyse versus analysed.

We have reviewed and addressed your recommendations.

The statistics represented in your tables are a bit confusing. Some refer to 1.6 times. Others represent what I presume are r scores. You have also used two different forms of parentheses (e.g. ( and [ ) in these tables, and I am unsure if that is due to differences in findings or a reporting error. You also report confidence intervals and I think standard errors, dependent on the study. Is there a reason for this? You have also omitted the interpretation of SE in your footnotes for these tables.

- Unfortunately the studies all reported their results differently, some with SE, others with p values
- [] replaced with () in tables
- SE interpretation added in footnote

The tables are also not in APA format. There are a number of formatting, referencing, grammatical and punctuation errors throughout that require attention. I have done my best to make more specific comments below that may help in this process, although I have likely missed some and gently recommend your attention with a more fine grained approach.

The tables have been changed to APA format, thank you for the specific comments and we have also reviewed the grammar, punctuation and references (these follow the Vancouver style referencing).

I understand this is sometimes challenging with multiple authors, so suggest the primary author to choose a tense, and referencing style, and go from there. Some examples are headings/bolding and italicising of text, table names and numbers.

We have reviewed the tense, referencing style and text style.

I also wonder why you have listed the statistics in the paragraphs and reiterated them again in the tables. This method is viewed unfavourably by reviewers and readers as it increases burden. I would instead suggest referring to the findings broadly in text, and referring to the tables for statistics. E.g. There were inconsistent results relating to …. for children/adolescents/offspring experiencing MI versus FI.. where X studies identified increased ….., while X studies identified decreased…. and X studies identified no association between Y and Z (see Table X).

You also can probably omit the authors name in that section as you list them in the table, this will shorten the manuscript and it will sound much more concise (and help interpretability of findings).

We have actioned these suggestions as far as is possible. As most studies are from the USA, we have retained authors’ names as to delineate one study from another as country where the study was conducted (so often used in reviews) was not possible here.

Specific Comments:

Line 9: ‘...wellbeing outcomes who children who experience…’ I think you may have meant to say wellbeing outcomes for children who.. - This has been changed.

Line 27: ‘Having either parent in prion..’ Correct spelling error, prison. This has been changed.

Line 52: Consider caregiving responsibility rather than caring responsibility This has been changed.

Line 69: ‘...being unable to see or talk can be extensive…’ - revise sentence, important to be specific. E.g. ‘unable to see or talk’ in this context would refer to seeing or talking to their incarcerated parent, not unable to see or talk generally. This has been changed.

Line 75: unnecessary use of : This has been changed.

Line 79: Omit ‘statistically’ This has been changed.

Line 80: Be specific, significantly associated with PTSD/mental health conditions for whom? The incarcerated parent or their offspring? This has been changed.

Line 94: revise this sentence. ‘..be taken into statutory care of grandparents’ is an incomplete sentence. E.g. ‘be taken into statutory or grandparental care’ This has been changed.

Line 95-96: Revise sentence and use of ‘individual separate’ This has been changed and revised.

Line 112: ‘process: included’ should this be ‘process included:’ ? This has been changed.

Line 143: Table 1 - Use of various font sizes, inconsistent punctuation. Repeated mention of checking review articles’ reference list. These have been revised.

Line 158-159: Check APA guidelines for use of written versus numerics for numbers 10 and above. E.g. sixty-nine versus 71 This has been changed.

Line 177: Counties or countries? Line 177: mortality of children or the incarcerated parent? Both have been changed.

Line 178: Parental reports of depression of their children or themselves? This has been changed.

Line 186: Revise this sentence. United States of American Adolescents.. ? This has been changed.

Line 190: Is the 15701 the sample at wave 4? If yes, indicate with n= This has been changed.

Line 191-192: You mention ‘in jail’ or ‘in prison’. If there is a difference in jail versus prison, you should elaborate in the introduction/method. If there is not a difference and you want to capture both terms, use incarcerated or imprisoned, just to not confuse the reader This has been changed to imprisoned.

Line 210-214: You have previously established MI versus FI, so use those terms consistently. You begin discussing no parental imprisonment earlier in the manuscript, so it would be better to define this term (NPI) earlier and use consistency throughout. This has been changed.

Line 218: Make sure statistics are represented in APA format (i.e. italicised where required) This has been changed.

Line 234: ‘AddHealth’ should there be a space? This has been changed.

Line 235: Revise sentence ‘as demonstrated by two separate studies showed that’ This has been changed.

Line 238: ‘Geller et al’ - this is not APA and it’s one of the first times you have mentioned et al. with an intext reference. Please ensure you have this consistent throughout, usually in-text references also require publication year in parenthesis. This has been addressed as per the Vancouver referencing style which does not have the publication year in the test for in-text citations.

You also inconsistency use a period after et al. throughout this and preceding and succeeding paragraphs. The use of a period after et al has been changed to ensure it is consistent throughout.

Line 252: use of experienced and experience in the same sentence. Recommend going through the paper and correcting all to be in past tense. This sentence has been changed and the tense reviewed.

Line 254: may be helpful to define ‘acting-in’ - I presume this opposes acting-out, but may be helpful to clarify This sentence has been changed as acting in is internalising feelings.

Table 3: Please check the statistics you are representing. Some of the CI’s have a zero before the period, some do not. Some of the papers have the statistic represented in parenthesis (e.g. CI or SE) while others do not, yet still have statistics reported. Please ensure you are determining the statistical test you are referencing. It is difficult to compare across studies with different statistical tests represented, so ensure you are being clear about what is reported and list all of the terms in the footnote (e.g. t-test, OR, SE, Beta)

These have been checked and modified as appropriate.

It is also unclear whether you are referring to reports on offspring. For example, parent self-report suggests that the mother or father is reporting on their own mental health experience, whereas using parent-report would indicate their report on their child. Self-report suggests child reporting on their own mental health etc.

This has been changed and we have clarified these details.

Line 280: There is a sentence that is in parenthesis but is separated from the previous sentence by a period. - This has been changed and we have clarified these details.

Line 303-305: statistics need additional parentheses and parenthesis within a parenthesis requires use of square brackets: i.e. (aOR 1.7, 95% CI[1.1−2.8]). Statistics listed in line 304 are not in parenthesis while those in line 305 are. This occurs again on line 309. This has been changed.

Line 306: You did not define STI in the above line before using the abbreviation. This has been changed and defined.

Table 4: Is there a difference between the reference to ‘use’ versus ‘abuse’. In this table you indicate cocaine, marijuana, or substance use, and then prescription drug abuse. This has been changed.

Also maybe move the Table 4 outcomes to under the substance and alcohol use section, and keep Table 5 under diet, etc. Thank you, this has been changed.

Line 335: ‘Indeed…..same study population’. Revise this sentence. This has been revised.

Line 353: ‘experiencing parental imprisonment…impact their children’s health and wellbeing’. If you are referring to the child experiencing parental imprisonment it impacts their health and wellbeing, not their children’s. This has been addressed.

Line 356-357: Reference? The reference has been added.

Line 363: atomised? Maybe inconsistent or incongruent? The wording has been changed.

Line 364: reviewing the impact maternal and paternal imprisonment on children. Has on children? The impact of maternal and paternal… etc. Revise The wording has been revised.

Line 364-365: I think this is implied. You could instead say, ‘this is a first attempt to synthesise existing literature relating to maternal and paternal imprisonment to gain a holistic understanding of the parental-gender impacts of incarceration on their child, both physically and psychologically’. This wasn’t an attempt, you did a great job with the literature you had - ensure you emphasise that this highlights an existing deficit within the literature that has failed to examine the potential risk for child health outcomes dependent on whether they experience a mother or father caregivers incarceration, compared to non-imprisoned parents. The wording has been changed.

Line 366: Very casual language. Revise. Avoid saying ‘very variable’ or ‘from how’. Use terms like methodological limitations that precluded meaningful interpretation, or a lack of consistency in definitions and measurements throughout the literature pertaining to parental incarceration and child outcomes. The wording has been changed to address the casual language.

Line 363-381: This section is a bit repetitive. Start by outlining the issues broadly, then use a sentence for each concern to specify the issue and why it is an important limitation for your study. Don’t be afraid to break limitations down to themes, i.e. ‘Methodological limitations’, ‘Measurement outcome limitations’, etc. This section has been revised and moved as appropriate to the strengths and weaknesses sections.

Line 382-388: Parent-reports or child mental health are also extremely incongruent to child self-reported mental health. There is a lot of subjectivity to consider, if a parent experiences their own mental health issue/substance or alcohol abuse issue, they are less likely to be acutely aware of physical or mental health issues in their child. Further, depression, anxiety, self harm and suicidality are extremely challenging for parents to be cognisant of, which adds another level of subjectivity and bias. There may also be a bit of social desirability bias, where parents where one or more caregivers have experienced imprisonment may want to avoid sharing the depth of detrimental impacts on their children, and report more favourable outcomes. These are just some considerations. This section has been revised.

Line 420: Consider another possibility that hypersexuality is rarely considered for youth as often there is a limited understanding of consent. It is possible that there is inconsistency in the age of investigation in early sexual behaviour due to the grey area between early sexuality and sexual abuse. Try your best when critiquing the inconsistency in the literature to propose other possibilities rather than methodological differences, which you have already touched on. Instead consider social and environmental impacts, as well as age of consent, lack of boundary setting from parents who have been incarcerated, poor parental monitoring, externalising behaviours, etc. Thank you, this section has been revised and wider social and environmental impacts on health and wellbeing have been included.

Line 451: You say you have limited understanding of the implications, but then say despite the substantial implications. Are you referring to the mechanism by which parental imprisonment is implicated in poorer mental health and physical wellbeing. If yes, say that explicitly. The wording has been revised.

Line 456: Reference? The reference has been added.

Line 458-461: This is a bit of a scoping claim. Make sure you support it with references, or if it is your opinion, say ‘although this interpretation is speculative’ or something along those lines. The wording has been revised.

Line 464: A lot of what you state in the strengths and weaknesses have been mentioned earlier in the discussion. Make sure you synthesise the discussion to ensure it reads better and is less repetitive.

The early part of the discussion section which considers issues relating to strengths and weaknesses has now been moved to that section.

Line 511-518: I think your conclusion is lacking the overarching take away. While it is evident that parental incarceration may place children at risk of detrimental physical and psychological outcomes, there is still limited understanding of the differences that may occur when it is a mother versus a father that is imprisoned. Further research is necessary to understand the potential similarities and differences based on parental sex, and the influence this has on child functioning … etc

The concluding paragraph has been developed further.
